# MiR-22-3p suppresses sepsis-induced acute kidney injury by targeting PTEN

**DOI:** 10.1042/BSR20200527

**Published:** 2020-06-02

**Authors:** Xudong Wang, Yali Wang, Mingjian Kong, Jianping Yang

**Affiliations:** 1 Department of Anesthesiology, Affiliated First Hospital, Soochow University, Suzhou, China; 2 Department of Anesthesiology, the Second Affiliated Hospital of Xuzhou Medical University, Xuzhou, China

**Keywords:** acute kidney injury, miR-22-3p, nflammation, PTEN, sepsis

## Abstract

**Background:** Septic acute kidney injury is considered as a severe and frequent complication that occurs during sepsis. The present study was performed to understand the role of miR-22-3p and its underlying mechanism in sepsis-induced acute kidney injury.

**Methods:** Rats were injected with adenovirus carrying miR-22-3p or miR-NC in the caudal vein before cecal ligation. Meanwhile, HK-2 cells were transfected with the above adenovirus following LPS stimulation. We measured the markers of renal injury (blood urea nitrogen (BUN), serum creatinine (SCR)). Histological changes in kidney tissues were examined by hematoxylin and eosin (H&E), Masson staining, periodic acid Schiff staining and TUNEL staining. The levels of IL-1β, IL-6, TNF-α and NO were determined by ELISA assay. Using TargetScan prediction and luciferase reporter assay, we predicted and validated the association between PTEN and miR-22-3p.

**Results:** Our data showed that miR-22-3p was significantly down-regulated in a rat model of sepsis-induced acute kidney injury, *in vivo* and LPS-induced sepsis model in HK-2 cells, *in vitro*. Overexpression of miR-22-3p remarkably suppressed the inflammatory response and apoptosis via down-regulating HMGB1, p-p65, TLR4 and pro-inflammatory factors (IL-1β, IL-6, TNF-α and NO), both *in vivo* and *in vitro*. Moreover, PTEN was identified as a target of miR-22-3p. Furthermore, PTEN knockdown augmented, while overexpression reversed the suppressive role of miR-22-3p in LPS-induced inflammatory response.

**Conclusions:** Our results showed that miR-22-3p induced protective role in sepsis-induced acute kidney injury may rely on the repression of PTEN.

## Introduction

Sepsis, as the leading cause of death in organ dysfunction, is thought to be associated with infections caused by bacteria, virus or fungi, causing a systemic inflammatory response syndrome [1,2]. As the most common complication of sepsis, acute kidney injury is diagnosed by elevated serum creatinine (SCR) and blood urea nitrogen (BUN) [3], which significantly decreases the ability of the kidney to filter blood and prolongs the stay in the intensive care unit [4]. It is estimated that septic patients with different degrees of damage (51% septic shock, 23% severe and 19% moderate) are diagnosed as sepsis-induced acute kidney injury [5,6]. Current specific therapies, including renal replacement have made some advances, but the refractory outcomes and higher mortality rate are still reported [7,8]. Therefore, exploring the underlying molecular mechanisms will help to develop and improve the therapies for this disease.

PTEN (Phosphatase and tensin homolog deleted on chromosome 10) consists of 9 exons and encodes protein-containing 403 amino acids with phosphatase activity [9]. PTEN functions as an important regulator in PI3K/protein kinase B (AKT) signaling pathway for inflammatory diseases [10,11] and is also responsible for angiogenesis, cell survival and other biological processes [12]. Importantly, accumulating evidence has indicated the crucial role of PTEN in sepsis-induced diseases. For example, Yang et al. [13] demonstrated that miR-30a-3p overexpression could improve sepsis-induced cell apoptosis in H9C2 cells by PTEN-mediated-PI3K/AKT signaling pathway. Yao et al. [14] also demonstrated that miR-25 reduced LPS-induced cardiomyocyte apoptosis by down-regulating PTEN/TLR4/NF-κB axis. Increase in the rate of autophagy via PTEN/AKT/mTOR pathway has been reported to be involved in valproic acid attenuating sepsis-induced myocardial dysfunction in rats [15]. In addition, HMGB1/PTEN/β-catenin signaling is a novel pathway that regulates the regulatory T-cell (Treg) development and provides a potential therapeutic target in sepsis-induced lung injury [16]. However, the upstream regulatory mechanisms underlying PTEN involved in sepsis-induced acute kidney injury has not been fully understood.

Recent studies demonstrated microRNAs (miRNAs/miRs), as endogenous non-coding RNA molecules are important mediators in degrading mRNA or inhibiting translation of their target genes to act as crucial molecular markers in diagnosis and prognosis of acute kidney injury [17,18]. By searching for related studies, we found that most of studies focused on the role of miRNAs in ischemic acute kidney injury, while its role in sepsis-induced acute kidney injury is limited [19], including miR-21 [20], miR-204 [21], miR-290-5p [22] and miR-106a [23]. Interestingly, a recent study by Ge et al. [24] reported the markedly decreased miR-22-3p expression in sepsis-induced kidney injury. Interestingly, PTEN was a potential target of miR-22-3p in different diseases, including renal cell carcinoma [25], diabetic nephropathy [26] and cisplatin chemosensitivity of gastric cancer [27]. Nevertheless, the role of miR-22-3p and its association with PTEN in sepsis-induced acute kidney injury still remain unclear.

In the present study, we developed a sepsis-induced acute kidney injury rat model, *in vivo*, by cecal ligation and puncture and LPS-induced sepsis model, *in vitro*, in HK-2 cells. Then, we analyzed the expression levels of miR-22-3p and PTEN. Next, we investigated the biological function of miR-22-3p and PTEN in inflammatory response and apoptosis by performing gain-of-function, loss-of-function and rescue experiments. The association between miR-22-3p and PTEN was further confirmed by luciferase reporter assay. Our findings might provide a theoretical basis for the development of sepsis-induced acute kidney injury and its treatment.

## Materials and methods

### Model construction and animal groups

Male Sprague-Dawley (SD) rats (180–220 g, 8 weeks) were purchased from Shanghai SLAC Laboratory Animal Co. Ltd. (Shanghai, China) and kept in an environment with 50 ± 10% humidity at 21–23°C under a 12 -h light–dark cycle. All rats had free access to food/water before the experiments. Rats were randomly divided into the following four groups (*n*=5 per group): Sham group, Model group, Model + adenovirus carrying miR-NC (Ad-miR-NC) group and Model + Ad-miR-22-3p group. The sepsis model group was constructed by cecal ligation and puncture (CLP) as described previously [28,29]. Briefly, about 1/3 of the cecum was ligated with a 5-0 suture, and punctured twice with a 21G needle. Then, gently squeezed to make a small amount of intestinal contents overflow from the puncture hole to ensure patency. Following ligation, rats were placed onto a heating pad to maintain the temperature at 37 ± 0.5°C. The sham group underwent cecum isolation without ligation of puncture. Before CLP operation, the rats from the caudal vein Model + Ad-miR-NC and Model + Ad-miR-22-3p groups were injected with Ad-miR-NC or Ad-miR-22-3p (GenePharma Co., Ltd., Shanghai, China). All experiments were performed by anesthetizing rats with 3% sodium pentobarbital, killed by carbon dioxide asphyxiation, and performed under the guidance of professionals at the Experimental Animal Center of Soochow University. All experimental protocols were approved by the Animal Ethics Committee of Affiliated First Hospital Soochow University and strictly performed in accordance with international ethical guidelines and the National Institutes of Health Guide concerning the Care and Use of Laboratory Animals.

### Sample preparation

At 48 h following surgery, rats from different groups were anesthetized with 3% sodium pentobarbital and then killed. We utilized an aortic puncture to collect blood samples for quantification of serum creatinine (SCR) and blood urea nitrogen (BUN). On the other hand, the kidney tissues were immediately collected following anesthetization. After washing with PBS, some of tissues were frozen in liquid nitrogen for quantitative real-time PCR and Western blot analysis, and the other tissues were fixed in 4% paraformaldehyde overnight and embedded in paraffin for histological examination.

### Histological examination

Paraffin-embedded kidney tissues were cut into 4-μm-thick sections and prepared for a series of histological evaluation. For Hematoxylin and Eosin (H&E) staining, the sections were deparaffinized using xylene and stained with hematoxylin for 5 min at room temperature. After being washed, the sections were incubated with Eosin for 2 min at room temperature and observed for the histological morphology under a light microscope (200× or 400× magnification). For Masson staining, we stained the sections for 5 min with 1% Hematoxylin, washed twice with 95% ethanol, stained with acid ponceau solution for 1 min, and observed them under an optical microscope (200× or 400× magnification). For PAS staining, the sections were deparaffinized with xylene, stained with PAS (both from Sigma-Aldrich) and then analyzed under a light microscope (200× or 400× magnification). For TUNEL assay, the sections were de-waxed and dehydrated. After removing the endogenous peroxidase activity, the sections were incubated with TUNEL reaction liquid for 1 h at 37°C. Subsequently, 2% DAB developing solution was used to visualize the sections for 15 min at room temperature under a fluorescence microscope.

### Cell culture and transfection

Human tubular epithelial cell line HK-2 (CRL-2190) was purchased from American type culture collection (ATCC, Manassas, VA, U.S.A.) and cultured in RPMI-1640 medium supplemented with 10% FBS at 37°C with 5% CO_2_. HK-2 cells were treated with 10 μg/ml of lipopolysaccharide (LPS, Source: Escherichia coli 055: B5, #L8880, Solarbio, Beijing, China) for 24 h in cell culture medium. For cell infection, HK-2 cells were infected with Ad-miR-NC, Ad-miR-22-3p, sh-NC or sh-PTEN respectively, followed by LPS treatment. All viruses were purchased from GenePharma (Shanghai, China). The PTEN coding sequences were subcloned into pcDNA4.0 (Sangon Biotech, China) to construct pcDNA4.0-PTEN expression vectors. The pcDNA4.0-PTEN or empty pcDNA4.0 vector was transfected into Ad-miR-22-3p-infected HK-2 cells, followed by LPS stimulation in the rescue experiments. All transfection protocols were performed for 48 h with Lipofectamine 3000 (Thermo Fisher Scientific, Waltham, U.S.A.).

### ELISA assay

Serum was obtained by centrifugation of the blood samples at 3000 × ***g*** for 15 min at 4°C. Meanwhile, the cellular supernatant was collected and centrifuged at 1000 rpm for 20 min. The supernatant was collected for ELISA assay according to manufacturer’s instructions (R&D Systems, Inc. MN, U.S.A.). The concentration of IL-1β, IL6, TNF-α or NO was calculated by standard sample.

### Quantitative real-time PCR

RNA from kidney tissues or cells were extracted by TRIzol reagent (Invitrogen), precipitated in 75% ethanol and washed to extract total RNA. Reverse transcription was performed with SYBR primeScriptTM RT-PCR Kit (TaKaRa, Dalian, China). Then, qRT-PCR was conducted in accordance with the operation instructions of ExScriptTM RT-PCR Kit (TaKaRa, Dalian, China). The specific sequences of primers were shown in Table 1. The 2^−ΔΔCt^ method was used to calculate relative gene expression levels with U6 or GAPDH as the reference gene.

**Table 1:** Primers for quantitative real-time PCR

Gene name	Primer sequences (5′- 3′)
miR-22-3p	RT: CTCAACTGGTGTCGTGGAGTCGGCAATTCAGTTGAACAGTTCT
	F: ACACTCCAGCTGGGAAGCTGCCAGTTGAAGAA
U6	F: CTCGCTTCGGCAGCACA
	R: AACGCTTCACGAATTTGCGT
PTEN	F: GGAAAGGACGGACTGGTGTA
	R: CGCCTCTGACTGGGAATAGT
GAPDH	F: CCTCGTCTCATAGACAAGATGGT
	R: GGGTAGAGTCATACTGGAACATG

### Western blot analysis

Total protein samples were obtained using ice-cold radioimmune precipitation assay (RIPA) Lysis Buffer and quantified with a BCA protein kit (both from Beyotime Biotechnology, Shanghai, China). After 12% SDS-PAGE electrophoresis, separated protein samples were transferred to PVDF membranes, blocked with 5% skimmed milk for 2 h at room temperature, incubated with primary antibodies against PTEN, HMGB1, p65, p-p65, TLR4 and GAPDH overnight at 4°C, followed by next incubation with horseradish peroxidase (HRP)-conjugated secondary antibody for 2 h at room temperature. Next, the protein bands were visualized by enhanced chemiluminescence (GE Healthcare, Chicago, IL, U.S.A.).

### Luciferase reporter assay

Through TargetScan prediction, we identified the binding sites between PTEN and miR-22-3p. For luciferase reporter assay, the sequences covering the miR-22-3p target site in the wild-type (WT) and the mutant (MUT) 3′UTR of PTEN were cloned into the pGL3 luciferase vector (Promega, Madison, WI, U.S.A.). HK-2 cells were transfected with miR-22-3p or miR-NC and WT-PTEN or MUT-PTEN for 48 h using Lipofectamine 3000 (Thermo Fisher Scientific). The relative luciferase activities were determined by Dual-Luciferase Reporter Assay System kit (Promega).

### Statistical analysis

All experiments were repeated three times and corresponding data were expressed as mean ± SD. Statistical differences for two groups were performed with Student’s *t*-test and those for multiple groups with one-way analysis of variance (ANOVA), followed by a post hoc Tukey test. Values of *P* less than 0.05 were thought to be statistically significant.

## Results

### Up-regulation of miR-22-3p significantly attenuated sepsis-induced acute kidney injury *in vivo*

**Figure 1:**
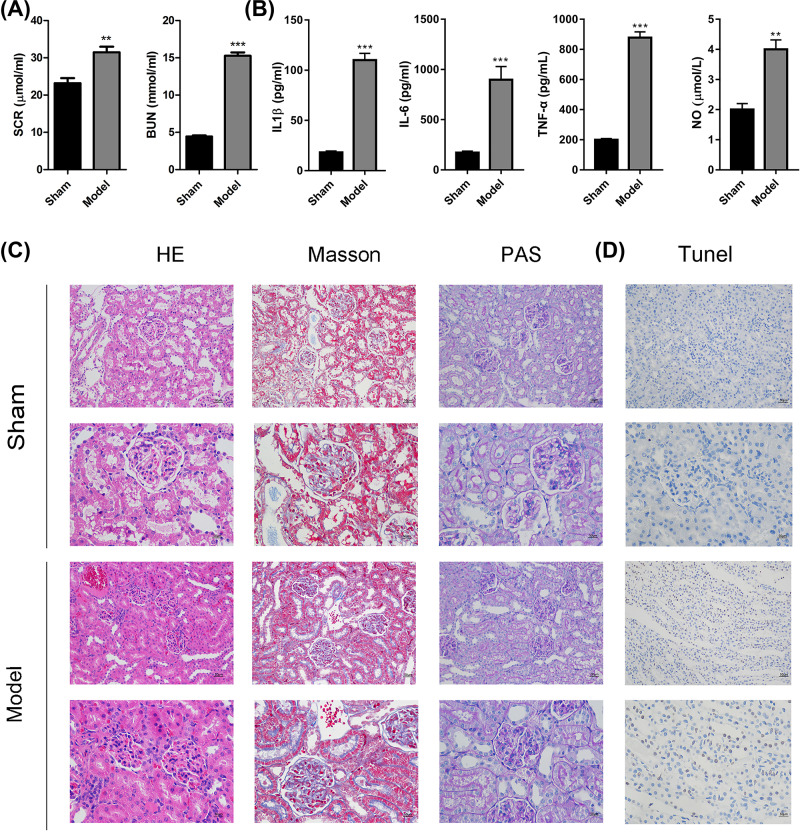
Establishing the sepsis-induced kidney injury rat model (**A**) At 48 h after surgery, rats were anesthetized and the blood samples were collected for quantification of SCR and BUN using a 7600 Automatic Biochemical Analyzer. (**B**) Serum was collected and the supernatant was used for ELISA assay. The concentrations of IL-1β, IL6, TNF-α or NO were calculated using a standard sample. (**C**) H&E staining was used to observe histological cell morphology and inflammatory changes. The cortex, including glomeruli, and renal interstitium are shown. Masson staining was applied to observe fibers and inflammatory factors in the interstitial lesions. A single glomerulus is shown. Periodic acid Schiff (PAS) staining was utilized to detect sugars in the glomerular mesangial area and the basement membrane are shown. (**D**) The apoptosis status was assessed using TUNEL assay. Arrows display Tunel positive cells. Data are expressed as mean ± SD; *N*=5, **P<0.01, ***P<0.001.

We first established the rat sepsis model and found that both the SCR and BUN expression levels were significantly enhanced in the septic rat models (Figure 1A). Additionally, the levels of serum IL-1β, IL-6, TNF-α and NO were significantly increased in model groups (Figure 1B). In the results from H&E, Masson and PAS (Figure 1C) staining, we observed that there was a small amount of fibrous hyperplasia in the glomerulus accompanied by inflammatory cell infiltration, significant glomerular swelling, and narrow or even occluded capillary cavity compressed by the proliferation and infiltration of cells in the model group when compared with the sham group. Moreover, TUNEL staining showed that sepsis-induced apoptosis in kidney tissues (Figure 1D). These data indicated that we successfully established an acute sepsis-induced kidney injury model in rats.

**Figure 2:**
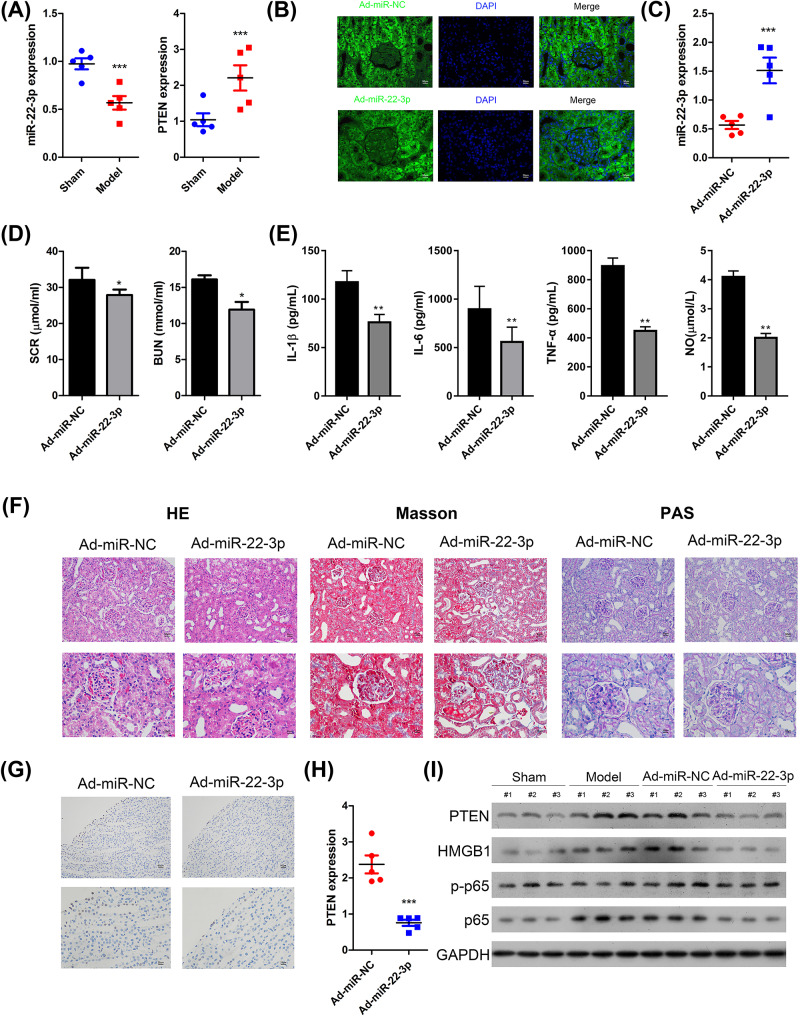
Effects of miR-22-3p overexpression on inflammatory response in the kidneys of sepsis-induced kidney injury rat models (**A**) The expression of miR-22-3p and PTEN was determined using quantitative real time PCR in kidney tissues. (**B**) Ad-miR-NC or Ad-miR-22-3p was injected through the caudal vein in sepsis model rats. After 48 h, the expression of GFP was observed in the kidney tissues. (**C**) The expression of miR-22-3p was determined using quantitative real time PCR in kidney tissues. (**D**) The expression levels of SCR and BUN were measured using a 7600 Automatic Biochemical Analyzer. (**E**) ELISA assay was performed to analyze the levels of IL-1β, IL-6, TNF-α and NO. (**F**) H&E staining was used to observe histological cell morphology and inflammatory changes. The cortex, including glomeruli, and renal interstitium are shown. Masson staining was applied to observe fibers and inflammatory factors in the interstitial lesions. A single glomerulus is shown. Periodic acid Schiff (PAS) staining was utilized to detect sugars in the glomerular mesangial area and the basement membrane are shown. (**G**) The apoptosis status was assessed using TUNEL assay. Arrows show Tunel positive cells. (**H**) The expression of PTEN was determined using quantitative real-time PCR in kidney tissues. (**I**) Western blot analysis was performed to detect the protein expression of PTEN, HMGB1, p65, p-p65 and TLR4 in kidney tissues. Data are expressed as mean ± SD; *N*=5, *P<0.05, **P<0.01, ***P<0.001.

To identify the effects of miR-22-3p on sepsis, we detected the expression of miR-22-3p in Sham operated and sepsis rat model and found that the expression of miR-22-3p was significantly reduced and the expression of PTEN was induced in the sepsis rat model (Figure 2A). Next, we overexpressed miR-22-3p expression by an injection of adenovirus carrying Ad-miR-22-3p via the caudal vein, to investigate the functional role of miR-22-3p in sepsis-induced acute kidney injury. By observing the GFP-positive signals, we confirmed adenovirus carrying Ad-miR-22-3p or Ad-miR-NC had successfully entered the kidney tissues and the expression of miR-22-3p was confirmed using real-time PCR (Figure 2B,C). As shown in Figure 2D,E, miR-22-3p overexpression significantly reduced the SCR and BUN expression levels and the levels of serum IL-1β, IL-6, TNF-α and NO were also significantly suppressed in the septic rat model. We additionally collected the kidney tissues and measured the expression of PTEN using quantitative real-time PCR. As illustrated in Figure 2F, injection of Ad-miR-22-3p significantly reduced the edema of the glomerular tissue cells, there was improvement in the fibrosis of the glomeruli, as well as the stenosis and occlusion of the renal lumen. Moreover, TUNEL staining additionally indicated that miR-22-3p overexpression effectively inhibited the sepsis-induced apoptosis in kidney tissues (Figure 2G). Furthermore, the mRNA expression of PTEN was reduced in the Ad-miR-22-3p injected rats (Figure 2H). Western blot analysis further confirmed that miR-22-3p overexpression significantly down-regulated the expression of PTEN, HMGB1, p-p65 and TLR4 in sepsis-induced acute renal injury rat models (Figure 2I). Based on these data, we concluded that miR-22-3p overexpression could improve the *in vivo* model of sepsis-induced acute kidney injury.

### Up-regulation of miR-22-3p significantly reduced the inflammatory response in LPS-induced sepsis model *in vitro*

**Figure 3:**
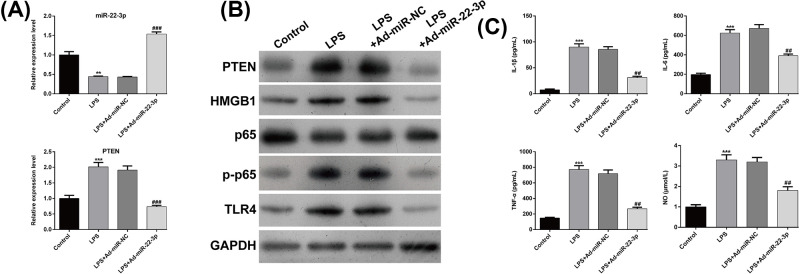
Up-regulation of miR-22-3p reduced the inflammatory response in LPS-induced sepsis-induced kidney injury model in vitro HK-2 cells were transfected with Ad-miR-NC or Ad-miR-22-3p, followed by LPS treatment. (**A**) The expression of miR-22-3p and PTEN was determined using quantitative real-time PCR. (**B**) Western blot analysis was conducted to detect the protein expression of PTEN, HMGB1, p65, p-p65 and TLR4. (**C**) ELISA assay was performed to analyze the levels of IL-1β, IL-6, TNF-α and NO. Data are expressed as mean ± SD.; **P<0.01, ***P<0.001, compared with control; ##*P*<0.01, ###*P*<0.001, compared with LPS + Ad-miR-NC.

Next, HK-2 cells were treated with LPS to mimic the *in vitro* septic model to confirm the protective role of miR-22-3p in sepsis-induced acute kidney injury. As shown in Figure 3A, miR-22-3p was down-regulated and PTEN was up-regulated in LPS group when compared with the control group, which were both reversed following miR-22-3p overexpression in LPS-stimulated HK-2 cells. Western blot analysis demonstrated that miR-22-3p overexpression significantly reduced the LPS-induced up-regulation of PTEN, HMGB1, p-p65 and TLR4 (Figure 3B). In addition, we analyzed the levels of inflammatory cytokines using ELISA assay. Consistent with the *in vivo* data, overexpression of miR-22-3p significantly attenuated LPS-induced increase of IL-1β, IL-6, TNF-α and NO in HK-2 cells (Figure 3C).

### PTEN is a direct target of miR-22-3p

**Figure 4:**
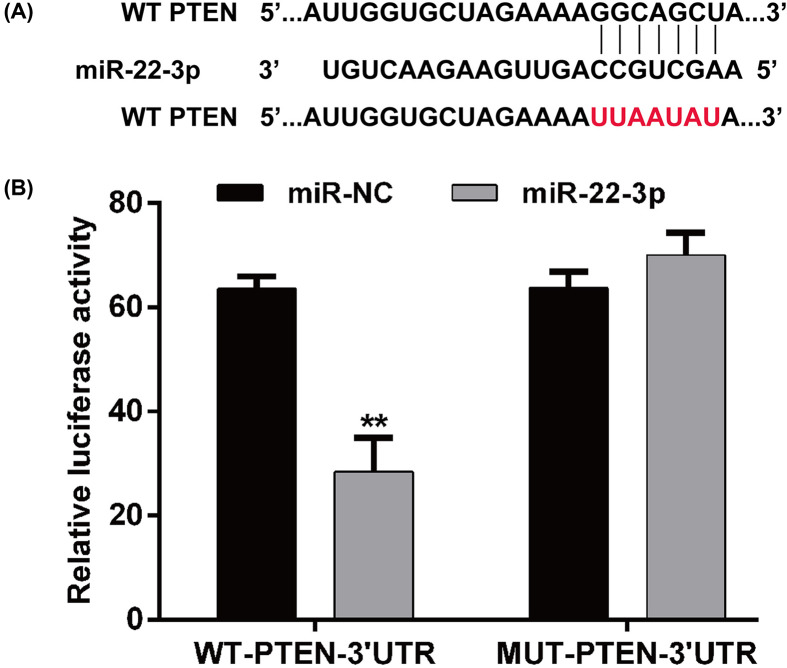
PTEN is a direct target of miR-22-3p (**A**) With TargetScan prediction, the fragment containing the specific binding site of miR-22-3p was found to be located in PTEN-3′-UTRs. (**B**) The correlation between PTEN and miR-22-3p was verified by dual-luciferase reporter system. Data are expressed as mean ± SD. **P<0.01, compared with miR-NC

The potential target of miR-22-3p was screened to further explore the mechanisms underlying miR-22-3p leading to improvement in sepsis-induced kidney injury model. Among the identified target genes, we selected PTEN as a potential target of miR-22-3p and their binding site is shown in Figure 4A. As shown in Figure 4B, miR-22-3p overexpression significantly decreased the luciferase activity of pGL3-PTEN-WT, but did not affect luciferase activity of pGL3-PTEN-MUT, significantly.

### MiR-22-3p attenuated the *in vitro* LPS-induced sepsis by targeting PTEN

**Figure 5:**
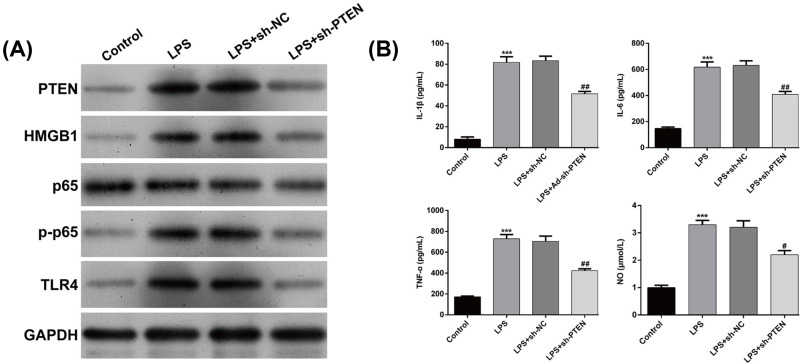
Knockdown of PTEN reduced the inflammatory response in LPS-induced sepsis-induced kidney injury model in vitro HK-2 cells were transfected with sh-NC or sh-PTEN, followed by LPS treatment. (**A**) Western blot analysis was conducted to detect the protein expression of PTEN, HMGB1, p65, p-p65 and TLR4. (**B**) ELISA assay was performed to analyze the levels of IL-1β, IL-6, TNF-α and NO. Data are expressed as mean ± SD. ***P<0.001, compared with control; #*P*<0.05, ##*P*<0.01, compared with LPS + sh-NC

**Figure 6:**
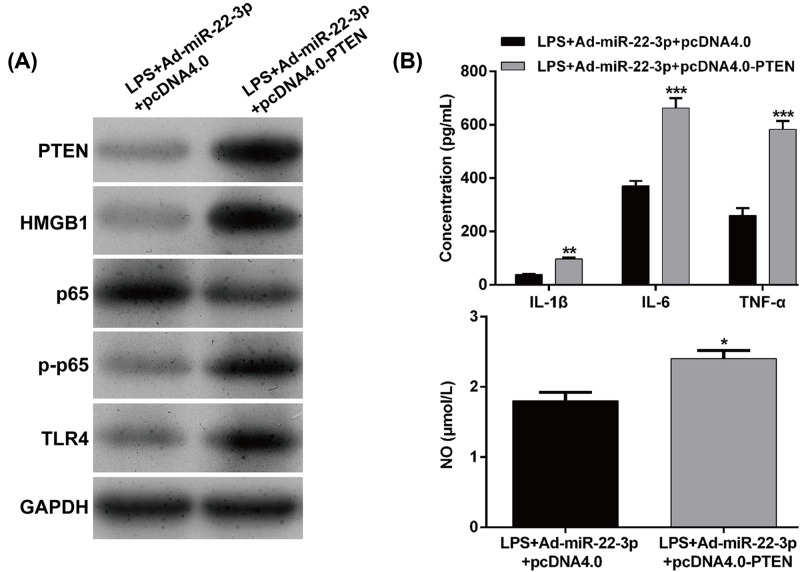
Overexpression of PTEN reversed the effects of miR-22-3p on the inflammatory response in LPS-induced kidney injury model in vitro HK-2 cells were co-transfected with Ad-miR-22-3p with pcDNA4.0 or pcDNA4.0-PTEN, followed by LPS treatment. (**A**) Western blot analysis was conducted to detect the protein expression of PTEN, HMGB1, p65, p-p65 and TLR4. (**B**) ELISA assay was performed to analyze the levels of IL-1β, IL-6, TNF-α and NO. Data are expressed as mean ± SD. *P<0.05, **P<0.01, ***P<0.001, compared with LPS + Ad-miR-22-3p + pcDNA4.0.

To confirm whether PTEN was a downstream functional regulator involved in miR-22-3p regulation of LPS-induced sepsis model *in vitro*, we first performed loss-of-function assays in LPS-stimulated HK-2 cells by transfection with sh-PTEN or sh-NC. As shown in Figure 5A, Western blot analysis confirmed PTEN was significantly decreased following sh-PTEN transfection. Under PTEN knockdown condition, we found that the LPS-induced expression of HMGB1, p-p65 and TLR4 was significantly inhibited in HK-2 cells. Similar to miR-22-3p overexpression, PTEN knockdown significantly alleviated the increased expression of IL-1β, IL-6, TNF-α and NO in HK-2 cells (Figure 5B). Furthermore, we performed rescue experiments by transfecting pcDNA4.0-PTEN or empty pcDNA4.0 into Ad-miR-22-3p-transfected HK-2 cells following LPS stimulation. As demonstrated by Western blot, pcDNA4.0-PTEN transfection significantly up-regulated PTEN expression, which was accompanied by an increased expression of HMGB1, p-p65 and TLR4 (Figure 6A). ELISA assay consistently demonstrated that PTEN overexpression significantly elevated the levels of IL-1β, IL-6, TNF-α and NO (Figure 6B). Collectively, these data demonstrated that miR-22-3p could decrease LPS induced attenuation of the inflammatory response, *in vitro* by targeting PTEN.

## Discussion

In the present study, we found that miR-22-3p was significantly down-regulated sepsis-induced acute kidney injury, both *in vivo* in a rat model and *in vitro* in LPS-induced HK-2 cells. Overexpression of miR-22-3p significantly suppressed the inflammatory response and cell apoptosis, both *in vivo* and *in vitro*, by targeting PTEN. Our results illustrated that miR-22-3p exerted a protective role in sepsis-induced acute kidney injury.

miR-22-3p has been shown to play a suppressive role in inflammatory cytokine pathway in dust mite induced asthma attack [30], the microenvironment of colonic neoplasms [31] and chronic obstructive pulmonary disease [32]. A previous study has been found that the expression of has-miR-22-3p was significantly decreased in patients with sepsis when compared with healthy volunteers [24]. A recent study showed that a long noncoding RNA, TCONS_00016233 drives sepsis-induced acute kidney injury by targeting miR-22-3p/AIFM1 pathway [33]. Here, we have demonstrated that in an *in vivo* sepsis-induced acute kidney injury rat model, the expression of miR-22-3p was reduced significantly. Additionally, overexpression of miR-22-3p significantly reversed the pathological changes and inflammatory factors in acute kidney injury model, indicating that miR-22-3p played a protective role in acute kidney injury. However, a recent study found that the inhibition of miR-22-3p *in vivo* resulted in decreased IgG deposition in the kidney, decreased STAT1 phosphorylation and decreased kidney disease, in a mouse model of systemic lupus erythematosus (SLE) (doi: https://doi.org/10.1101/512848). The contradicting results may have been caused by different animal models.

LPS is a classical ligand for TLR4 and mediates TLR4-dependent signal transduction to activate NF-κB, leading to an increase in inflammatory cytokine expression [34,35]. LPS-induced human proximal tubule cell line (HK-2 cells) was served as an *in vitro* model of sepsis-induced acute kidney injury in several studies [36–38]. Additionally, the expression of TNF-α, IL-6, IL-8, IL-1β and MCP-1 mRNA and protein were significantly up-regulated in LPS-induced HK-2 cells [36,37]. In the present study, we confirmed the protective role of miR-22-3p *in vitro* by demonstrating the inhibitory effect on both inflammation and cell apoptosis by targeting PTEN.

Studies showed that PTEN was directly regulated by several miRNAs involved in proinflammatory cytokines production, such as miR-718 [39], miR-92a [40] and miR-26b [41]. HMGB1/PTEN/β-catenin signaling, as a novel pathway, plays an important role in sepsis-induced lung injury [16]. MiR-205 alleviates sepsis-induced renal injury through the HMGB1-PTEN signaling pathway [42]. In addition, miR-22-3p/PTEN axis plays a regulatory role in renal cell carcinoma [25], diabetic nephropathy [26] and cisplatin chemosensitivity of gastric cancer [27]. We showed that PTEN was a direct target of miR-22-3p and miR-22-3p down-regulated PTEN to suppress LPS-induced inflammation. These evidences further suggested that PTEN was a downstream functional regulator involved in miR-22-3p regulation of sepsis-induced renal injury.

HMGB1, as a downstream mediator of sepsis, has been previously demonstrated by its regulatory role in inflammatory response and apoptosis [43], which is closely associated with different organ injuries [44–46]. Notably, Shen et al. [47] reported that miR-22/HMGB1 pathway was involved in HOTAIR accelerating sepsis-induced kidney injury. In addition, HMGB1 could promote the production of proinflammatory cytokines by regulating TLR2 or TLR4, causing the activation of coagulation and microvascular thrombosis [48]. Moreover, HMGB1/NF-κB signaling has been widely reported in inflammation-associated acute injuries, including spared nerve injury [49], acute lung injury [50] and cerebral ischemia–reperfusion injury [51]. We demonstrated that miR-22-3p overexpression reduced the expression of p-65 and TLR4, accompanied by the inhibition of HMGB1, suggesting that miR-22-3p may reduce inflammatory response and apoptosis, in sepsis-induced kidney injury rat model and LPS induced cell injury model, through HMGB1/NF-κB pathway.

In conclusion, the *in vitro* and *in vivo* data demonstrated that miR-22-3p overexpression may exhibit a beneficial effect by attenuating sepsis-induced or LPS-induced inflammation and apoptosis, by targeting PTEN. These data suggest that miR-22-3p/PTEN might be a promising therapeutic target for sepsis-induced acute kidney injury.

## Abbreviations

BUN: blood urea nitrogen
H&E: hematoxylin and eosin
LPS: lipopolysaccharide
PTEN: phosphatase and tensin homolog deleted on chromosome 10
SCR: serum creatinine
SLE: systemic lupus erythematosus
Treg: regulatory T-cell
